# Influenza A Virus (H1N1) Infection Induces Glycolysis to Facilitate Viral Replication

**DOI:** 10.1007/s12250-021-00433-4

**Published:** 2021-09-14

**Authors:** Lehao Ren, Wanju Zhang, Jing Zhang, Jiaxiang Zhang, Huiying Zhang, Yong Zhu, Xiaoxiao Meng, Zhigang Yi, Ruilan Wang

**Affiliations:** 1 Department of Emergency and Critical Care Medicine, Shanghai General Hospital, Shanghai Jiao Tong University School of Medicine, Shanghai, 201620 China; 2 Department of Critical Care Medicine, Union Hospital, Tongji Medical College, Huazhong University of Science and Technology, Wuhan, 430022 China; 3 Microbiology Laboratory, Shanghai Municipal Centre for Disease Control and Prevention, Shanghai, 200336 China; 4 Department of Pathology, Zhongshan Hospital, Fudan University, Shanghai, 200032 China; 5 Department of Pathogen Diagnosis and Biosafety, Shanghai Public Health Clinical Center, Fudan University, Shanghai, 201508 China

**Keywords:** H1N1, Glycolysis, Replication, Hypoxia-inducible factor 1 (HIF-1), Interferon

## Abstract

Viruses depend on host cellular metabolism to provide the energy and biosynthetic building blocks required for their replication. In this study, we observed that influenza A virus (H1N1), a single-stranded, negative-sense RNA virus with an eight-segmented genome, enhanced glycolysis both in mouse lung tissues and in human lung epithelial (A549) cells. In detail, the expression of hexokinase 2 (HK2), the first enzyme in glycolysis, was upregulated in H1N1-infected A549 cells, and the expression of pyruvate kinase M2 (PKM2) and pyruvate dehydrogenase kinase 3 (PDK3) was upregulated in H1N1-infected mouse lung tissues. Pharmacologically inhibiting the glycolytic pathway or targeting hypoxia-inducible factor 1 (HIF-1), the central transcriptional factor critical for glycolysis, significantly reduced H1N1 replication, revealing a requirement for glycolysis during H1N1 infection. In addition, pharmacologically enhancing the glycolytic pathway further promoted H1N1 replication. Furthermore, the change of H1N1 replication upon glycolysis inhibition or enhancement was independent of interferon signaling. Taken together, these findings suggest that influenza A virus induces the glycolytic pathway and thus facilitates efficient viral replication. This study raises the possibility that metabolic inhibitors, such as those that target glycolysis, could be used to treat influenza A virus infection in the future.

**Supplementary Information:**

The online version contains supplementary material available at 10.1007/s12250-021-00433-4.

## Introduction

Viruses are obligate intracellular parasites that depend on host cellular metabolism to accomplish their replication. An array of diverse viruses induces dramatic alterations in host cellular metabolic pathways including glycolysis, fatty acid synthesis, and glutaminolysis for their benefits (Sanchez and Lagunoff 2015). Although there are similarities among virus-induced metabolic modifications to some extent, each virus species may require unique metabolic alterations to complete its life cycle (Sanchez and Lagunoff 2015). For example, white spot syndrome virus, dengue virus, norovirus, and Kaposi’s sarcoma herpesvirus (KSHV) induce glycolysis (Chen *et al*. 2011; Yogev *et al*. 2014; Fontaine *et al*. 2015; Passalacqua *et al*. 2019), whereas hepatitis C virus and vaccinia virus activate glutamine catabolism (Fontaine *et al*. 2014; Thai *et al*. 2015; Asim *et al*. 2017; Levy *et al*. 2017). Moreover, tomato bushy stunt virus (TBSV) recruits pyruvate kinase into its replicase complex to generate ATP to fuel its own replication (Chuang *et al*. 2017).

Influenza virus, a member of the *Orthomyxoviridae* family with a genome composed of eight single-stranded, negative-sense RNA segments, causes substantial morbidity and mortality annually, becoming a large threat to public health (Iuliano *et al*. 2018). However, influenza virus has evolved strategies to evade host immune response and to escape from vaccine protection. It has been reported that neuraminidase inhibitors, the current frontline anti-influenza drugs, have been met with a rise in viral resistance (Hurt 2014; Li *et al*. 2015). Therefore, it is imperative to develop drugs that target host cellular machinery (Zumla *et al*. 2016). Cellular metabolism related host factors are promising targets against influenza (Muller *et al*. 2012).

Glucose is mainly catabolized through two pathways, glycolysis and the tricarboxylic acid (TCA) cycle. It has been reported that many viruses can reprogram glucose metabolism in the host cells (Thai *et al*. 2014; Yogev *et al*. 2014; Yu *et al*. 2014; Fontaine *et al*. 2015; Passalacqua *et al*. 2019; Zhao *et al*. 2019). Activation of glycolysis by influenza A virus has been reported by some studies (Genzel *et al*. 2004; Ritter *et al*. 2010; Petiot *et al*. 2011; Smallwood *et al*. 2017). However, the cell lines and specimens used in these studies were still not adequate. In this study, we show that glycolysis is upregulated in H1N1-infected human lung epithelial (A549) cells and mouse lung tissues and that inhibiting this metabolic pathway results in reduced H1N1 replication. Furthermore, the effect of glycolytic inhibitors or enhancer on H1N1 infection is independent of interferon signaling. According to these findings, H1N1 infection activates the glycolytic pathway of glucose metabolism to support efficient viral replication.

## Materials and Methods

### Cells and Viruses

Human lung adenocarcinoma epithelial (A549) cells and Madin Darby Canine Kidney (MDCK) cells were purchased from the ATCC. A549 cells were cultured in Ham’s F-12 K medium supplemented with 10% FBS and 1% antibiotics (100 U/mL penicillin and 0.1 mg/mL streptomycin). MDCK cells were cultured in DMEM medium supplemented with 10% fetal bovine serum (FBS) and 1% antibiotics. All cells were cultured in a humidified atmosphere with 5% CO_2_ at 37 °C. The influenza A/PR/8/34 strain was purchased from the ATCC. A549 cells were seeded and, after incubation overnight, were washed with PBS twice and then infected with influenza virus at the indicated multiplicity of infection (MOI). After 2 h of adsorption, the inoculum was removed, and the cells were maintained in maintenance medium (Ham’s F-12 K containing 25 mmol/L HEPES, 1% antibiotics, and 0.25 μg/mL TPCK-treated trypsin) for the indicated time.

### Animal Model

Female BALB/c mice, at 4–6 weeks old, were obtained from the Chinese Academy of Sciences Experiment Center in Shanghai. The mice were intranasally infected with influenza A virus (A/PR/8/34) at 800 PFU/mouse or 50 μL of saline. After 3 d, blood was collected and serum was harvested by centrifugation. Then the mice were sacrificed, and the mouse lung tissues were rapidly collected and cryopreserved in liquid nitrogen.

### Reagents

2-deoxyglucose (2DG, catalog number D8375) and oxamate (catalog number O2751) were purchased from Merck (St. Louis, MO, USA). Dichloroacetate (DCA; catalog number S8615) was purchased from Selleck Chemicals (Houston, TX, USA). PS48 (catalog number HY-15967) was purchased from MedChemExpress (Monmouth Junction, NJ, USA). 2DG, oxamate, DCA and PS48 were directly solubilized in cell culture medium at indicated concentrations. Ham’s F-12 K medium and FBS were purchased from Gibco/BRL Life Technologies (Grand Island, NY, USA). Anti-HIF-1α (catalog number SAB2702132) primary antibody was purchased from Merck (St. Louis, MO, USA). Anti-PKM2 (catalog number 4053), anti-HK2 (catalog number 2867), anti-GAPDH (catalog number 5174), and anti-β-actin (catalog number 4970 s) primary antibodies were purchased from Cell Signaling Technology (Boston, MA, USA). Anti-H1N1 influenza A virus nucleocapsid (NP; catalog number ab104870) primary antibody was purchased from Abcam (Cambridge, MA, USA). Anti-PDK3 (catalog number H00005165-M01) was purchased from Novus Biologicals (Littleton, CO, USA). Anti-HIF-1β (catalog number 611078) was purchased from BD Transduction Laboratories (Franklin Lakes, NJ, USA). The respective horseradish peroxidase (HRP)-conjugated secondary antibodies were obtained from Beyotime (Shanghai, China). The radio immunoprecipitation assay (RIPA) protein lysis buffer, phenylmethanesulfonyl fluoride (PMSF), bicinchoninic acid (BCA) protein concentration assay kit, and SDS-PAGE gel preparation kit were obtained from Beyotime (Shanghai, China). PVDF membranes and highly sensitive enhanced chemiluminescence (ECL) agents were purchased from Bio-Rad (Richmond, CA, USA) and Thermo Fisher Scientific (Waltham, MA, USA), respectively. TRIzol was purchased from Invitrogen (Grand Island, NY, USA). A PrimeScriptTM RT Master Mix kit and a SYBR Premix Ex TaqTM II kit were purchased from TaKaRa (Dalian, Liaoning, China). A lactate assay kit was purchased from Merck (St. Louis, MO, USA). An ATP assay kit was purchased from Beyotime (Shanghai, China).

### Measurement of ATP Level

Intracellular ATP levels were measured by an ATP assay kit according to the manufacturer’s instructions. Briefly, the cells were lysed with ATP assay buffer. Next, ATP assay buffer containing firefly luciferase and luciferin was added to the samples and standards at room temperature for 5 s, and then the relative light units (RLUs) were measured in a luminometer. The amount of ATP was normalized to the protein concentration of the samples, as determined by BCA protein assay kit. All samples and standards were assayed in triplicate.

### Measurement of Lactate

The lactate in mouse serum, in mouse lung tissue homogenate, and in A549 cell supernatant was measured by a lactate assay kit according to the manufacturer’s instructions. The mouse lung tissue was homogenized in 4 volumes of lactate assay buffer and centrifuged at 13,000 ×*g* for 10 min to create a soluble fraction. The soluble fraction of the lung tissue, the mouse serum, and the cell supernatant were deproteinized with a 10 kDa MWCO spin filter that removed lactate dehydrogenase. The samples and lactate standards were added in duplicate to 96 well plates, and then, 50 μL of master reaction mix was added to each well and incubated for 30 min at room temperature. The absorbance was measured at 570 nm. The amount of lactate in the samples was then determined based on the standard curve.

### Western Blot Analysis

Total protein from the mouse lung samples and the cultured cells was extracted with RIPA buffer. The protein concentrations were detected using a BCA protein assay kit. Then total protein samples were separated on an 10% SDS-PAGE and transferred onto a PVDF membrane. The membranes were blocked with 5% non-fat milk in Tris-buffered saline with Tween-20 (TBST) and incubated overnight at 4 °C with primary antibodies against HIF-1α (1:1000), HK2 (1:1000), PKM2 (1:1000), PDK3 (1:1000), NP (1:1000), HIF-1β (1:2000), GAPDH (1:1000), and β-actin (1:1000). After washing in TBST, the bands were incubated with HRP-conjugated goat anti-rabbit secondary antibody (1:1000) or goat anti-mouse secondary antibody (1:1000) at room temperature for 1 h. After washing in TBST again, the membrane bands were visualized with the ECL reagent according to the manufacturer’s instructions.

### RNAi

The shRNA sequences used for knocking down human HIF-1α and HIF-1β were 5′-GTGATGAAAGAATTACCGAATCTCGAGATTCGGTAATTCTTTCATCAC-3′ and 5′-GAGACAGCTTCCAACAGGTCTCGAGACCTGTTGGAAGCTGTCTC-3′ respectively. These sequences and non-targeting irrelevant sequence were subcloned into the lentivirus vector pLKO.1 plasmid respectively. Then, pLKO.1 plasmids were used together with packaging plasmids (pSPAX2 and pMD2.G) to cotransfect HEK293T cells. Lentiviral stocks harvested were used to infect the A549 cells, followed by cell selection through puromycin (1 μg/mL).

### RNA Quantification

Total RNA was extracted with TRIzol reagent following the manufacturer’s instructions. One to two μg total RNA was reverse transcribed using the PrimeScript™ RT Master Mix. Quantitative real-time PCR was performed using SYBR Premix Ex Taq™ II on the Vii7 system (ABI). Relative mRNA level and intracellular viral RNA in each individual sample were examined in triplicate, normalized to β-actin, and calculated using the 2^−ΔΔCT^ method (Schmittgen and Livak 2008). The specific primers used for qRT-PCR are listed in Supplementary Table S1.

### Cell Viability Assay

Cell viability was determined by using the Cell-Counting Kit-8 (Dojindo, Kyushu, Japan) according to the manufacturer’s instructions. Briefly, 8 × 10^3^ control shRNA, shHIF-1α, and shHIF-1β A549 cells were seeded in opaque-walled 96-well plates. 24 h later, 10 μL of CCK-8 reagent was added directly into each well and incubated with the cells for 1 h at 37 °C. The absorbance of each well was measured at 450 nm with a microplate reader (Bio-Rad Instruments, USA).

### Plaque Assay

Plaque assays were performed in six-well plates as previously described (Matrosovich *et al*. 2006). Briefly, MDCK cells were infected with serial tenfold dilutions of the virus supernatants in 1 × MEM for 1 h at 37 °C. Then, the cells were washed with PBS and covered with 1% agarose in 1 × MEM with 1 μg/mL TPCK-treated trypsin. After 2–3 days of incubation, the plates were stained with crystal violet, and the visible plaques were counted.

### Statistical Analysis

All the data were statistically analyzed using GraphPad Prism (version 6.0; San Diego, CA, USA). Measurement data are expressed as mean (SD). An unpaired two-tailed Student’s *t*-test was used for comparisons between two indicated groups. A value of *P* < 0.05 was considered significant.

## Results

### H1N1 Infection Induces Glycolysis *In vivo* and *In vitro*

**Fig. 1:**
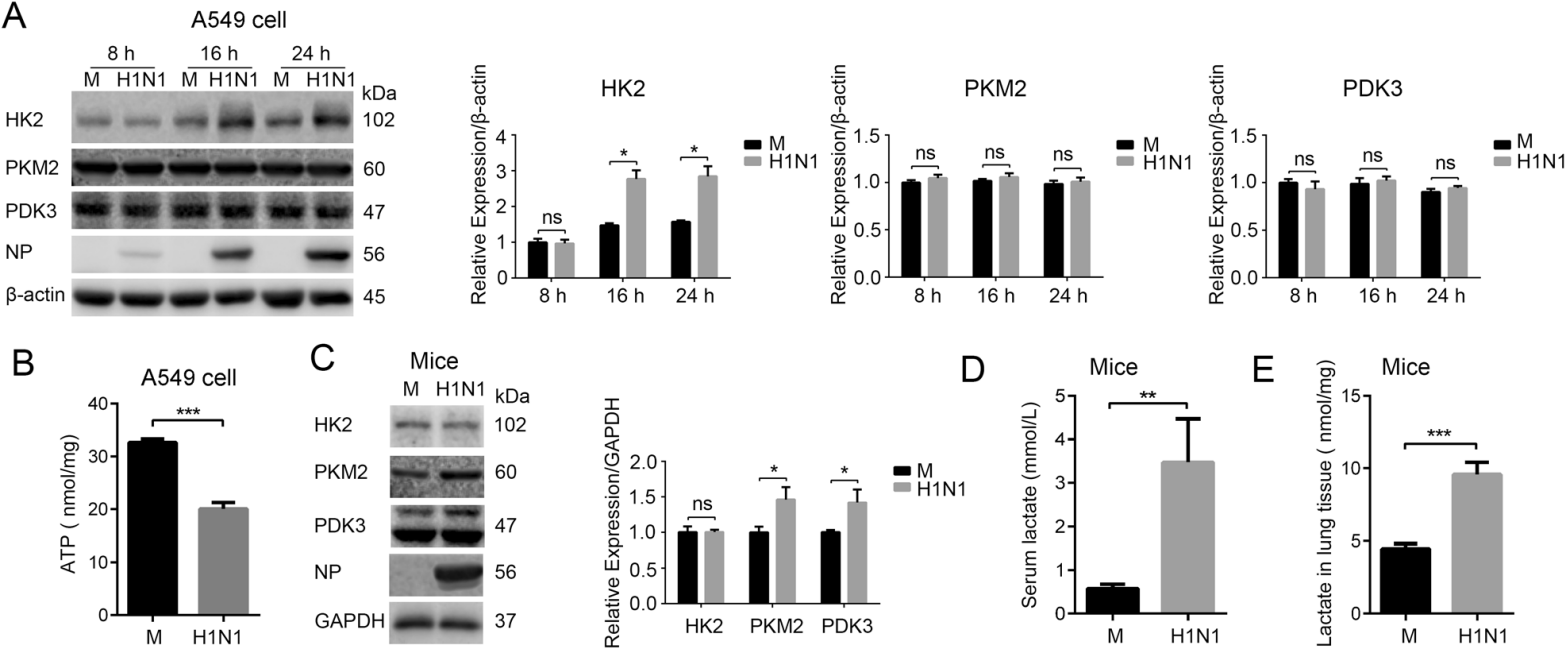
H1N1 infection induces glycolysis *in vivo* and *in vitro*. **A** A549 cells were mock infected or infected with H1N1 at an MOI of 1, and cells were harvested at 8, 16, and 24 h p.i.. The expression of HK2, PKM2, and PDK3 was analyzed by Western blotting. **B** A549 cells were mock infected or infected with H1N1 at an MOI of 1. Cells were harvested at 24 h p.i., and intracellular ATP levels were measured. **C**–**E** Mice were intranasally administered H1N1 (A/PR/8/34) 800 PFU/mouse or mock infected as controls (n = 3 mice/group). At 3 d p.i., the expression of HK2, PKM2, and PDK3 in mouse lung tissues was measured by western blotting (**C**) (the lower bands in PDK3 are nonspecific). Serum lactate (**D**) and lactate in lung tissue homogenate (**E**) were measured by a lactate assay kit. M, mock infection. ns, not significant. *P < 0.05, **P < 0.01, ***P < 0.001.

To identify the changes in glucose metabolism after H1N1 infection, we conducted a screen of key glycolytic enzymes in mock infected and H1N1 (A/PR/8/34) infected human lung epithelial (A549) cells, and we found that hexokinase 2 (HK2) was significantly upregulated after H1N1 infection at 16 h and 24 h post-infection (p.i.) (Fig. 1A). Hexokinase is the first rate-limiting enzyme in the glycolytic pathway, and the HK2 isoform, in particular, has been shown to be a key mediator of aerobic glycolysis (Wolf *et al*. 2011; Gershon *et al*. 2013). However, the expression of pyruvate kinase M2 (PKM2) and pyruvate dehydrogenase kinase 3 (PDK3) remained unchanged in the A549 cells after H1N1 infection (Fig. 1A). It is known that less ATP is produced when the same amount of glucose is oxidized by glycolysis than by TCA cycle, so we observed intracellular ATP levels in mock and H1N1-infected A549 cells at 24 h p.i.. As shown in Fig. 1B, intracellular ATP in H1N1-infected cells was significantly lower than that of mock infected cells, suggesting that glucose carbon was shunted away from the TCA cycle to glycolysis. To validate the influence of H1N1 infection on glucose metabolism, mice were intranasally administered H1N1 (800 PFU/mouse) or mock infected as a control. At 3 d p.i., the expression of PKM2 and PDK3 in H1N1-infected mouse lung tissues was significantly higher than that in mock infected group (Fig. 1C). Consistently, serum lactate and lactate in lung tissue homogenate in H1N1-infected group was significantly higher than that in mock infected group (Fig. 1D, 1E). Taken together, these results suggest that glycolysis is enhanced after H1N1 infection both *in vivo* and *in vitro.*

### Glycolytic Inhibitors Impair H1N1 Replication and Alleviate Virus-induced Cytopathy

**Fig. 2:**
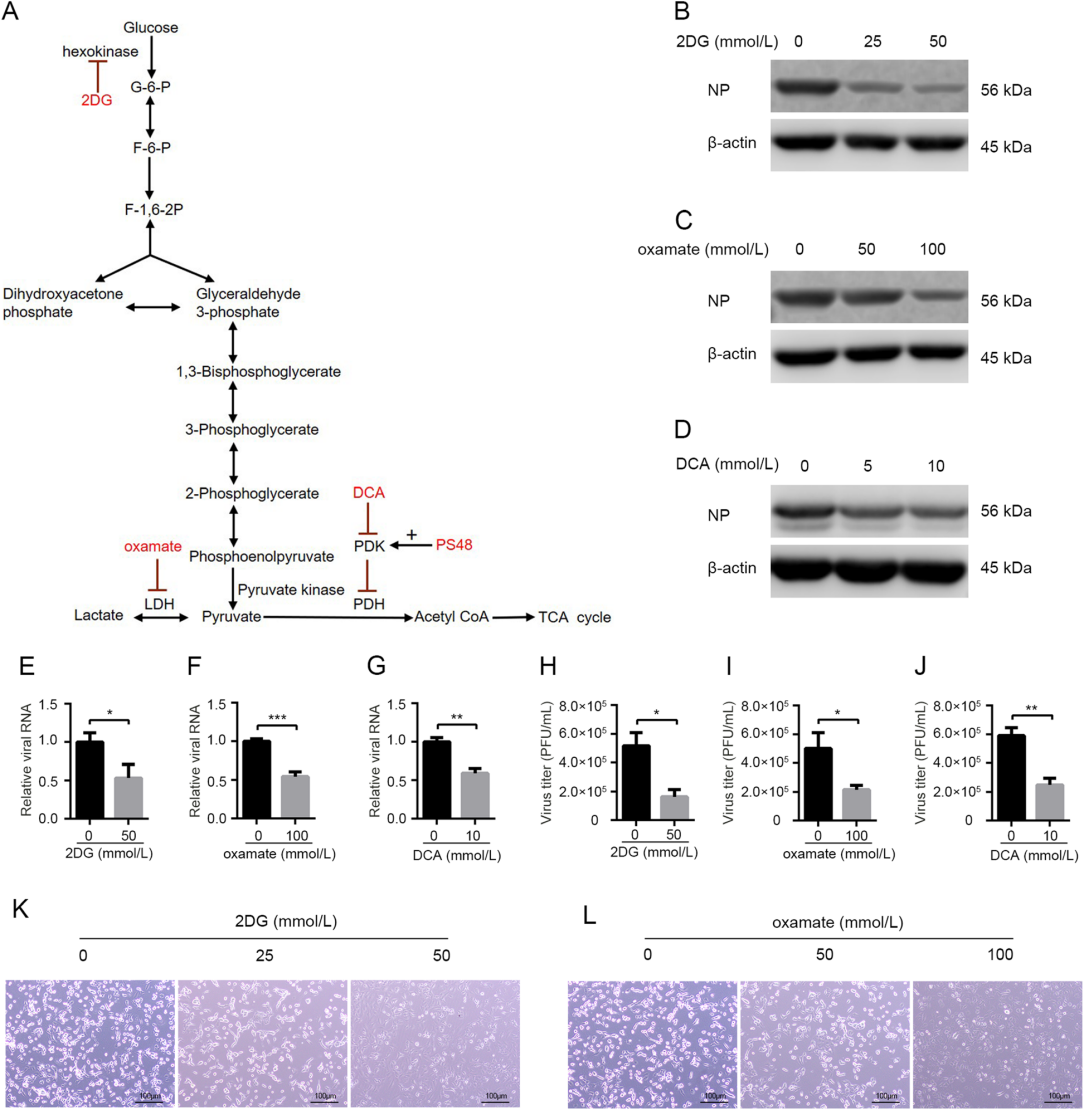
Glycolytic inhibitors impair H1N1 replication and alleviate virus-induced cell injury. **A** Schematic overview of the glucose metabolism and the functional targets of glycolytic inhibitors (2DG, oxamate, and DCA) and enhancer (PS48) used in this study. **B**–**L** A549 cells were infected with H1N1 at an MOI of 1, with or without glycolytic inhibitors treatment at the same time. **B**–**D** Cells were harvested at 24 h p.i., and intracellular NP levels were measured by Western blotting. **E**–**G** Cells were harvested at 24 h p.i., and intracellular viral RNA (*M*) levels were measured by qRT-PCR. β-actin expression was used as an internal control. **H**–**J** Cell supernatant were harvested at 24 h p.i., and viral titer levels were measured by plaque forming unit assay. **K, L** The morphological changes of A549 cells at 24 h p.i. under a phase contrast microscope were shown. *P < 0.05, **P < 0.01, ***P < 0.001.

As demonstrated above, glycolysis is activated in host cells after H1N1 infection. Some previous studies have reported that reprogrammed host cellular metabolism is beneficial for viral replication (Thai *et al*. 2014, 2015; Yu *et al*. 2014; Fontaine *et al*. 2015; Varanasi and Rouse 2018; Passalacqua *et al*. 2019). Then we examined whether enhanced glycolysis was required for H1N1 replication by using glycolytic inhibitors. 2DG, an analog of glucose and a competitive inhibitor of hexokinase, is a commonly used glycolysis inhibitor (Barban and Schulze 1961; Pelicano *et al*. 2006) (Fig. 2A**)**. A549 cells were infected with H1N1 at an MOI of 1, with or without different concentrations of 2DG treatment at the same time. At 24 h p.i., cells were harvested and intracellular influenza NP protein was determined by Western blotting. As shown in Fig. 2B, 2DG impaired H1N1 replication in a dose-dependent manner. To confirm the impact of glycolytic inhibitors on H1N1 replication, oxamate, a special lactate dehydrogenase inhibitor (Elwood 1968; Crane *et al*. 2014; Zhao *et al*. 2016) (Fig. 2A**)**, and DCA, which shifts glucose metabolism from glycolysis to TCA cycle by inhibiting pyruvate dehydrogenase kinase (Baker *et al*. 2000; Michelakis *et al*. 2008) (Fig. 2A), were also used in this H1N1-infected A549 cell model respectively. The results showed that H1N1-infected A549 cells treated with increasing concentrations of oxamate or DCA exhibited a dose-dependent reduction in viral replication (Fig. 2C, 2D). Consistently, intracellular viral RNA was significantly decreased upon 2DG, oxamate, and DCA treatment at 24 h p.i. (Fig. 2E–2G). Virus titer in cell supernatant was also significantly reduced upon 2DG, oxamate, and DCA treatment at 24 h p.i. (Fig. 2H–2J). Moreover, H1N1-induced cell injury was alleviated by 2DG and oxamate because of decreased viral replication (Fig. 2K, 2L).

### Glycolysis Inhibition by Knocking Down HIF-1 Pathway Impairs H1N1 Replication

**Fig. 3:**
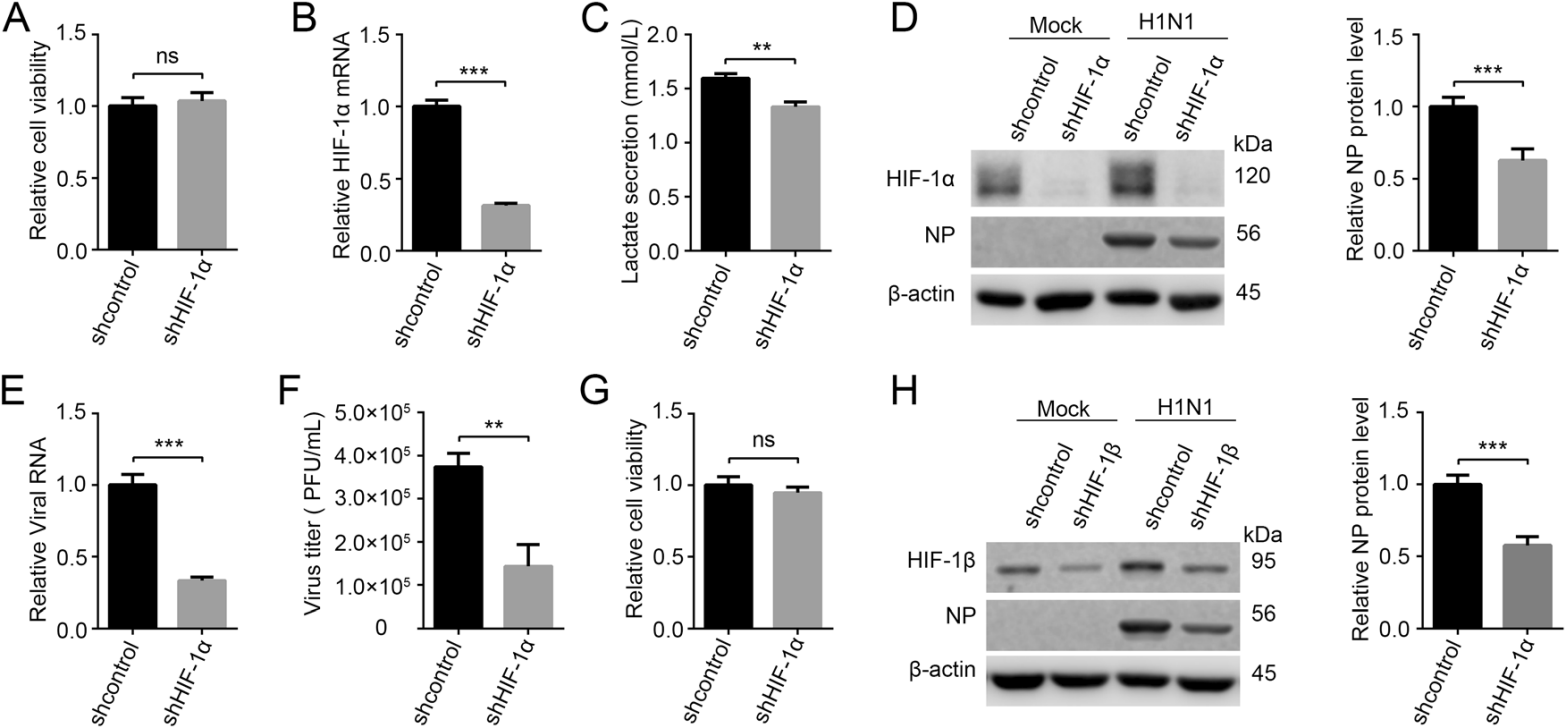
H1N1 replication is impaired by knocking down HIF-1 pathway. **A** Cell viability of shcontrol and shHIF-1α A549 cells was measured by a CCK-8 assay. **B, C** shcontrol and shHIF-1α A549 cells were infected with H1N1 at an MOI of 1, and cells and supernatants were harvested at 24 h p.i.. HIF-1α mRNA level was measured by qRT-PCR (**B**). Lactate in cell supernatant was measured by a lactate assay kit (**C**). **D**–**F** shcontrol and shHIF-1α A549 cells were mock infected or infected with H1N1 at an MOI of 1, and cells and supernatant were harvested at 24 h p.i.. The expression of HIF-1α and NP was analyzed by western blotting (**D**). Intracellular viral RNA (*M* gene) in infected groups was measured by qRT-PCR (**E**), β-actin expression was used as an internal control. Virus titer in cell supernatant in infected groups was measured by plaque forming unit assay (**F**). **G** Cell viability of shcontrol and shHIF-1β A549 cells was measured by a CCK-8 assay. **H** shcontrol and shHIF-1β A549 cells were mock infected or infected with H1N1 at an MOI of 1, and cells were harvested at 24 h p.i.. The expression of HIF-1β and NP was analyzed by western blotting. ns, not significant. **P < 0.01, ***P < 0.001.

It has been reported that HIF-1 upregulates glycolysis by activating the transcription of glycolytic enzymes and related regulatory enzymes such as HK2, PKM2, and PDK (Fulda and Debatin 2007; Semenza 2012; Xu *et al*. 2018). In our previous report, HIF-1 pathway was indeed upregulated after H1N1 infection (Ren *et al*. 2019). To further validate our finding that glycolysis is required for H1N1 replication, we downregulated host cell glycolysis by knocking down HIF-1 pathway and then examined its effect on H1N1 replication. Firstly, HIF-1α stable knocking-down (shHIF-1α) and control (shcontrol) A549 cell lines were established by lentivirus-mediated RNAi technology. HIF-1α downregulation had no significant impact on cell viability as measured by a CCK-8 assay (Fig. 3A), and HIF-1α mRNA and protein were significantly lower in shHIF-1α A549 cells compared to that in control cells (Fig. 3B, 3D). Besides, lactate concentration in H1N1-infected shHIF-1α A549 cell supernatant was significantly lower than that in H1N1-infected shcontrol A549 cell supernatant, indicating that glycolysis was indeed inhibited by knocking down HIF-1α (Fig. 3C). shcontrol and shHIF-1α A549 cells were mock-infected or infected with H1N1 at an MOI of 1, and the cells and supernatant were harvested at 24 h p.i.. The results showed that intracellular NP, intracellular viral RNA (*M* gene), and virus titers in supernatant were significantly lower in H1N1-infected shHIF-1α A549 cells than those in H1N1-infected shcontrol cells (Fig. 3D–3F). Notably, knocking down of HIF-1α in A549 cells did not inhibit the replication of enterovirus 71, indicating that the block in H1N1 replication was not due to the effects of HIF-1α knockdown on cell viability (Supplementary Fig. S1). To further validate our findings, we knocked down HIF-1β, the partner subunit of HIF-1α to form functional HIF-1 transcription factor (Wang and Semenza 1993, 1995), and then examined its impact on H1N1 infection. Consistently, HIF-1β knockdown had no effect on cell viability but significantly reduced H1N1 replication in A549 cells (Fig. 3G, 3H). Collectively, these data show that glycolysis is required for H1N1 replication.

### Pharmacologically Enhancing the Glycolytic Pathway Further Promotes H1N1 Replication

**Fig. 4:**
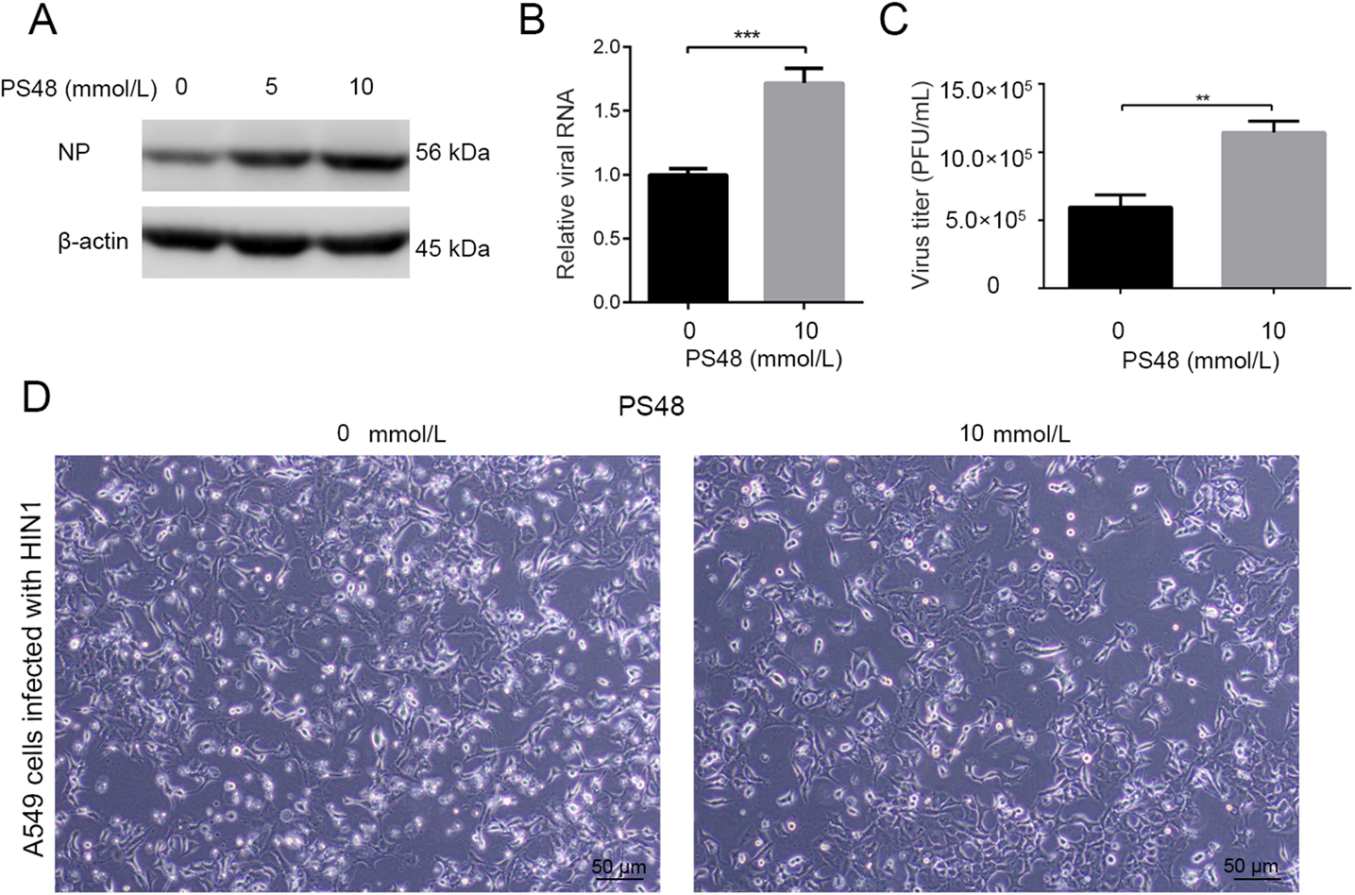
Pharmacologically enhancing the glycolytic pathway further promotes H1N1 replication. **A** A549 cells were infected with H1N1 at an MOI of 1, with or without PS48 (5 or 10 mmol/L) treatment at the same time. Cells were harvested at 24 h p.i., and intracellular NP level was measured by Western blotting. **B**, **C** A549 cells were infected with H1N1 at an MOI of 1, with or without PS48 (10 mmol/L) treatment at the same time. Cells were harvested at 24 h p.i., and intracellular viral RNA (*M*) was measured by qRT-PCR (**B**), β-actin expression was used as an internal control. Cell supernatants were harvested at 24 h p.i., and viral titer levels were measured by plaque forming unit assay (**C**). **D** A549 cells were infected with H1N1 at an MOI of 1, with or without PS48 (10 mmol/L) treatment at the same time. The morphological changes of A549 cells at 24 h p.i. under a phase contrast microscope were shown. **P < 0.01, ***P < 0.001.

To further validate the effect of host cell glycolysis on H1N1 replication, the effect of a glycolysis enhancer on H1N1 replication was determined. PS48, a PDK1 activator, shifts glucose metabolism from TCA cycle to glycolysis, inducing a Warburg-like metabolic state (Hindie *et al*. 2009; Han *et al*. 2017) (Fig. 2A). A549 cells were infected with H1N1 at an MOI of 1, with or without PS48 treatment at the same time. At 24 h p.i., cells and cell supernatant were harvested and intracellular NP protein, viral RNA, and virus titers in supernatant were examined by Western blotting, qRT-PCR, and plaque assay respectively. As shown in Fig. 4A–4C, PS48 promoted H1N1 replication by enhancing glycolysis. Moreover, H1N1 induced cell injury was aggravated by PS48 treatment due to boosted viral replication (Fig. 4D).

### The Effect of Glycolysis Inhibitors or Enhancer on Interferon Induction in H1N1-infected Cells

**Fig. 5:**
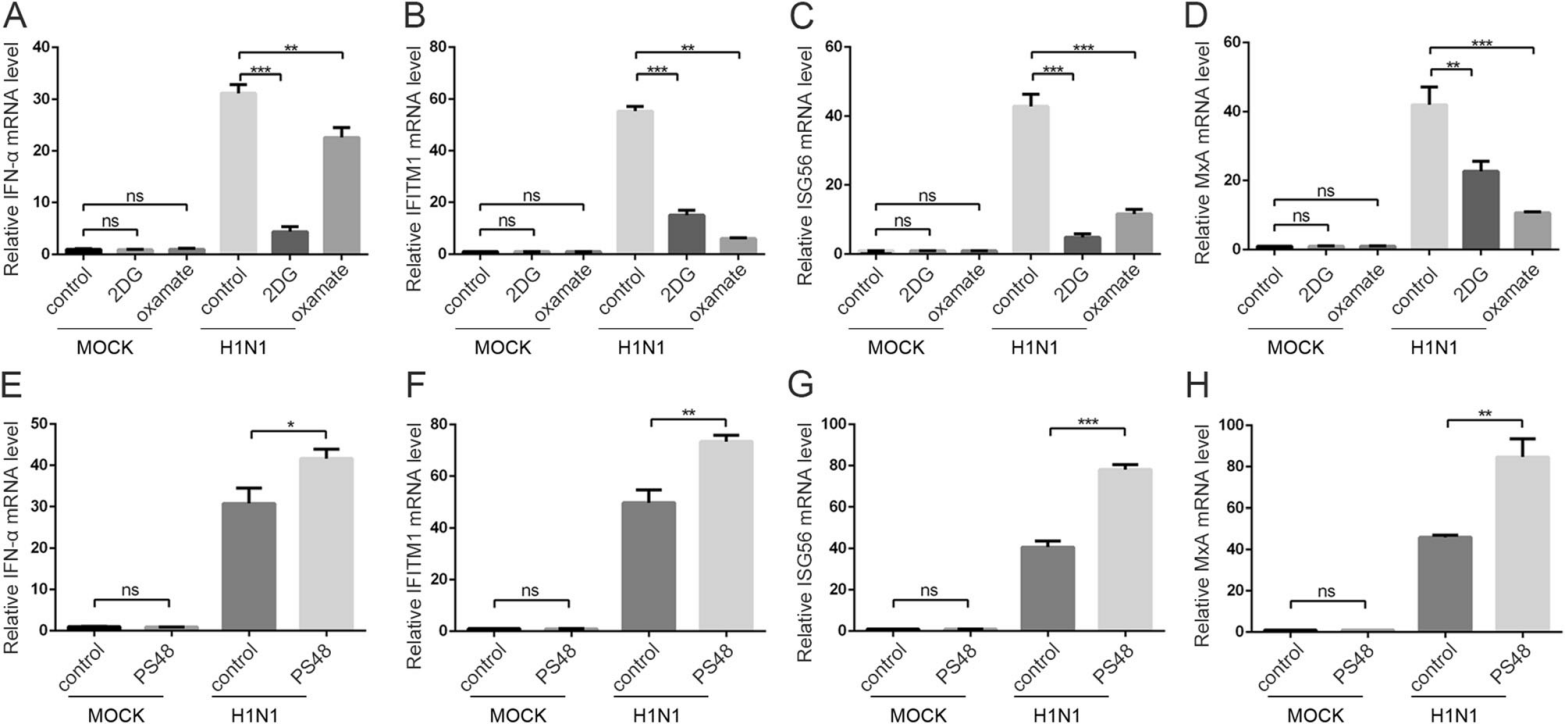
The effect of glycolysis inhibitors or enhancer on interferon induction. **A**–**D** A549 cells were mock infected or infected with H1N1 at an MOI of 1, with or without 50 mmol/L 2DG or 100 mmol/L oxamate treatment at the same time. Cells were harvested at 24 h p.i., and IFN-α (**A**), IFITM1 (**B**), ISG56 (**C**), and MxA (**D**) mRNA levels were measured by qRT-PCR. β-actin expression was used as an internal control. **E**–**H** A549 cells were mock infected or infected with H1N1 at an MOI of 1, with or without 10 mmol/L PS48 treatment at the same time. Cells were harvested at 24 h p.i., and *IFN-α* (**E**), *IFITM1* (**F**), *ISG56* (**G**), and *MxA* (**H**) mRNA levels were measured by qRT-PCR. β-actin expression was used as an internal control. **P < 0.01, ***P < 0.001.

Influenza infection activates innate immune response, inducing interferon production (Iwasaki and Pillai 2014; Herold *et al*. 2015), and influenza virus is sensitive to interferon treatment (Iwasaki and Pillai 2014). Recently, it was reported that lactate, the product of glycolysis, inhibits RIG-I like receptors-mediated interferon production (Zhang *et al*. 2019). As we demonstrated above that H1N1 infection prompted glycolysis, which may result in the inhibition of interferon (IFN) production because of the lactate-mediated inhibition of MAVS (Zhang *et al*. 2019). Therefore, we examined interferon production and interferon-stimulated genes (ISGs) in H1N1-infected A549 cells when glycolysis was blocked or enhanced by glycolytic inhibitors or enhancer. Strikingly, treatment with 2DG or oxamate did not rescue interferon production as expected, and the treatment resulted in significantly lower IFN-α, IFITM1, ISG56, and MxA transcription levels (Fig. 5A–5D). PS48 treatment resulted in significantly higher IFN-α, IFITM1, ISG56, and MxA transcription levels (Fig. 5E–5H). These results suggest that the change of H1N1 replication upon glycolysis inhibition or enhancement is independent of interferon signaling.

## Discussion

When influenza virus infects humans, it replicates rapidly in alveolar epithelial cells, and its rapid replication necessarily requires the organelles, energy, and biosynthetic substrates of host cells. So influenza virus has evolved sophisticated strategies to hijack host cells for their own benefits. For example, the viral ribonucleoprotein complex (vRNP) of influenza virus can preferentially utilize the newly generated RanGTP-CRM1 complex to complete the nuclear export process by taking dense chromatin as the carrier to obtain the spatial advantage with Ran guanine exchange factor Rcc1 (Chase *et al*. 2011). Besides this, host cellular metabolism may also be modulated by influenza virus for its replication. In this study, we examined glycolytic enzymes in human lung epithelial (A549) cells and mouse lung tissues after H1N1 infection. The results showed that HK2 was upregulated in A549 cells and that PKM2 and PDK3 were activated in mouse lung tissues after H1N1 infection. Lactate, the terminal product of glycolysis, was significantly elevated in H1N1-infected mouse serum and lung tissue homogenate. These results clearly indicate that glycolysis is activated in H1N1 infection. Some previous studies have also reported glycolysis is activated by influenza A virus infection (Genzel *et al*. 2004; Ritter *et al*. 2010; Petiot *et al*. 2011; Smallwood *et al*. 2017). These studies investigated influenza induced metabolism change in MDCK cells, HEK293 cells, and NHBE (normal primary human bronchial epithelial) cells by mass spectrometry, which were different from our study. H1N1 activates different glycolytic enzymes between human derived A549 cells and mouse lung tissues, and these differences may be due to different host genetic backgrounds. Besides, various cell types in the lung tissues, such as epithelial cells, endothelial cells, fibroblast, and immune cells, may contribute to this difference. The expression of PKM2 in mouse lung tissues is upregulated after influenza infection, which is consistent with a previous report (Miyake *et al.* 2017). In this report, PKM2 can interact with viral RNA-dependent RNA polymerase to promote virus replication (Miyake *et al*. 2017), which indicates another role of glycolytic enzyme in viral life cycle.

As demonstrated above, glycolysis is activated by influenza. However, glycolysis activation is not a common phenomenon in all viruses. For example, vaccinia virus does not induce or require glycolysis for replication (Fontaine *et al*. 2014). In addition to glucose metabolism, viruses can also modulate glutamine metabolism (Fontaine *et al*. 2014; Thai *et al*. 2015; Asim *et al*. 2017; Levy *et al*. 2017) or lipid metabolism (Koyuncu *et al*. 2013; Hsieh *et al*. 2015) of the host cell to promote self-replication. In other words, each virus may require unique metabolic changes in the host to achieve its own rapid replication (Sanchez and Lagunoff 2015). In this study, we focused on host cellular glucose metabolism, whether glutamine or lipid metabolism were also reprogrammed by influenza will be studied in the future.

Previous studies have reported that some other viruses enhance glycolysis of host cells to promote virus replication. However, the mechanisms of glycolysis activation are diverse. The gene product of adenovirus E4ORF1 binds to MYC, a transcription factor that upregulates energy metabolism through direct activation of metabolic genes, and thus enhances MYC binding to glycolytic target genes, resulting in elevated expression of specific glycolytic enzymes (Thai *et al*. 2014). Human cytomegalovirus robustly induces carbohydrate-response element binding protein (ChREBP) to reprogram glucose metabolism to enhanced glycolysis (Yu *et al*. 2014). Alphavirus YXXM motif in the viral nsP3 protein binds to PI3K regulatory subunit p85 and then activates cellular glycolysis (Mazzon *et al*. 2018). However, the mechanism of influenza induced glycolysis activation is still unclear. In our previous report, influenza A virus stabilizes HIF-1α via inhibition of proteasome (Ren *et al*. 2019). Glycolysis and viral replication were decreased after knocking down HIF-1, indicating that H1N1 induced glycolysis was dependent, at least in part, on HIF-1 pathway.

Using glycolytic enzyme inhibitors and enhancer in H1N1-infected A549 cell model, we found that enhanced glycolysis is indeed required for influenza replication. However, the detailed mechanism of glycolysis’s role in viral replication is still unknown. It has been reported that lactate, the end product of glycolysis, is a natural suppressor of retinoic-acid-inducible gene I like receptors (RLRs)-mediated interferon production (Zhang *et al*. 2019). However, our results showed that glycolysis inhibitors impair H1N1 replication with a reduced activation of interferon pathway and that glycolysis enhancer promotes viral replication with a boosted activation of interferon pathway, indicating that the effect of glycolysis on H1N1 replication may be independent of interferon signaling. It has been reported that the inhibition of murine norovirus replication by 2DG is independent of type I interferon (Passalacqua *et al*. 2019), which is similar to the situation in influenza infection. We speculate that activated glycolysis may provide more biosynthetic building blocks for viral RNA and protein synthesis so as to facilitate viral replication, independent of interferon pathway. In future studies, we will treat IFN receptor knockout cell lines or animal models with or without glycolysis inhibitors and then examine viral replication, whether the effect of glycolysis on viral replication is dependent on interferon will be clearly demonstrated.

**Fig. 6:**
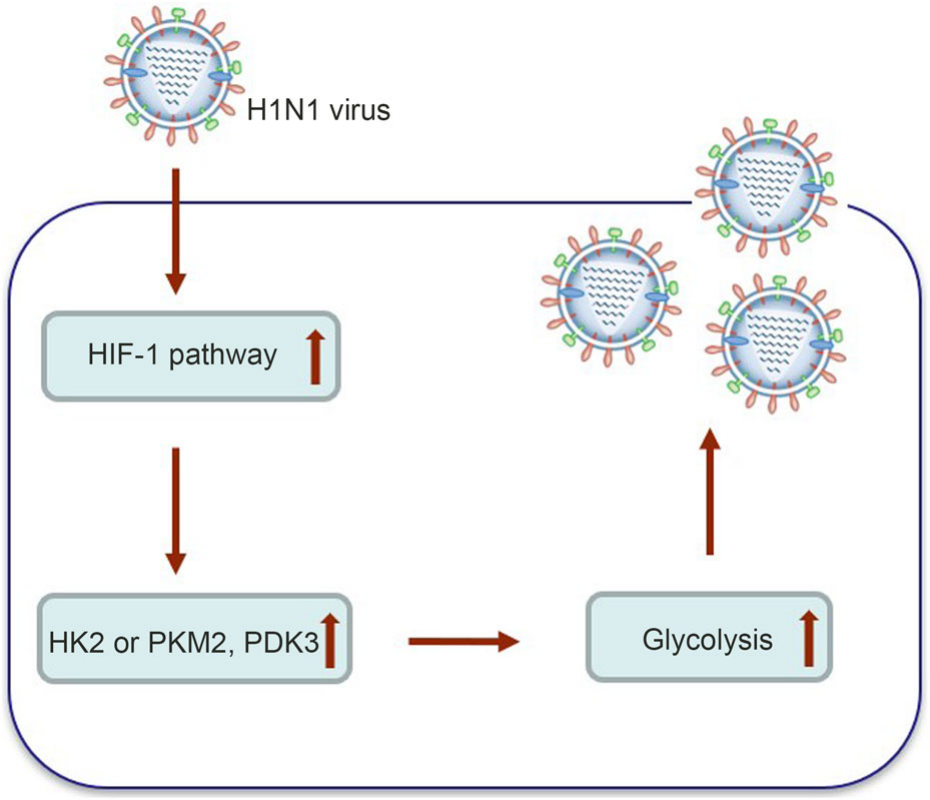
Schematic of the proposed model for glycolysis involvement in H1N1 replication. H1N1 activates HIF-1 pathway and then induces glycolysis to promote efficient viral replication.

In summary, the infection of alveolar epithelial cells by influenza A (H1N1) virus can activate HIF-1 signaling pathway, promote glycolysis of host cells, and provide support for the rapid replication of the virus (Fig. 6). Inhibition of glycolysis or HIF-1 pathway may provide a new direction for the treatment of influenza A (H1N1) infection.

## Supplementary Information

Below is the link to the electronic supplementary material.

Supplementary file1 (PDF 146 KB)

## References

[CR1] Asim M, Jiang S, Yi L, Chen W, Sun L, Zhao L, Khan Khattak MN, Tu J, Lin L (2017). Glutamine is required for red-spotted grouper nervous necrosis virus replication via replenishing the tricarboxylic acid cycle. Virus Res.

[CR2] Baker JC, Yan X, Peng T, Kasten S, Roche TE (2000). Marked differences between two isoforms of human pyruvate dehydrogenase kinase. J Biol Chem.

[CR3] Barban S, Schulze HO (1961). The effects of 2-deoxyglucose on the growth and metabolism of cultured human cells. J Biol Chem.

[CR4] Chase GP, Rameix-Welti MA, Zvirbliene A, Zvirblis G, Gotz V, Wolff T, Naffakh N, Schwemmle M (2011). Influenza virus ribonucleoprotein complexes gain preferential access to cellular export machinery through chromatin targeting. PLoS Pathog.

[CR5] Chen IT, Aoki T, Huang YT, Hirono I, Chen TC, Huang JY, Chang GD, Lo CF, Wang HC (2011). White spot syndrome virus induces metabolic changes resembling the warburg effect in shrimp hemocytes in the early stage of infection. J Virol.

[CR6] Chuang C, Prasanth KR, Nagy PD (2017). The Glycolytic Pyruvate Kinase Is Recruited Directly into the Viral Replicase Complex to Generate ATP for RNA Synthesis. Cell Host Microbe.

[CR7] Crane CA, Austgen K, Haberthur K, Hofmann C, Moyes KW, Avanesyan L, Fong L, Campbell MJ, Cooper S, Oakes SA, Parsa AT, Lanier LL (2014). Immune evasion mediated by tumor-derived lactate dehydrogenase induction of NKG2D ligands on myeloid cells in glioblastoma patients. Proc Natl Acad Sci U S A.

[CR8] Elwood JC (1968). Effect of oxamate on glycolysis and respiration in sarcoma 37 ascites cells. Cancer Res.

[CR9] Fontaine KA, Camarda R, Lagunoff M (2014). Vaccinia virus requires glutamine but not glucose for efficient replication. J Virol.

[CR10] Fontaine KA, Sanchez EL, Camarda R, Lagunoff M (2015). Dengue virus induces and requires glycolysis for optimal replication. J Virol.

[CR11] Fulda S, Debatin KM (2007). HIF-1-regulated glucose metabolism: a key to apoptosis resistance?. Cell Cycle.

[CR12] Genzel Y, Behrendt I, Konig S, Sann H, Reichl U (2004). Metabolism of MDCK cells during cell growth and influenza virus production in large-scale microcarrier culture. Vaccine.

[CR13] Gershon TR, Crowther AJ, Tikunov A, Garcia I, Annis R, Yuan H, Miller CR, Macdonald J, Olson J, Deshmukh M (2013). Hexokinase-2-mediated aerobic glycolysis is integral to cerebellar neurogenesis and pathogenesis of medulloblastoma. Cancer Metab.

[CR14] Han F, Xue M, Chang Y, Li X, Yang Y, Sun B, Chen L (2017). Triptolide suppresses glomerular mesangial cell proliferation in diabetic nephropathy is associated with inhibition of PDK1/Akt/mTOR pathway. Int J Biol Sci.

[CR15] Herold S, Becker C, Ridge KM, Budinger GR (2015). Influenza virus-induced lung injury: pathogenesis and implications for treatment. Eur Respir J.

[CR16] Hindie V, Stroba A, Zhang H, Lopez-Garcia LA, Idrissova L, Zeuzem S, Hirschberg D, Schaeffer F, Jorgensen TJ, Engel M, Alzari PM, Biondi RM (2009). Structure and allosteric effects of low-molecular-weight activators on the protein kinase PDK1. Nat Chem Biol.

[CR17] Hsieh YC, Chen YM, Li CY, Chang YH, Liang SY, Lin SY, Lin CY, Chang SH, Wang YJ, Khoo KH, Aoki T, Wang HC (2015). To complete its replication cycle, a shrimp virus changes the population of long chain fatty acids during infection via the PI3K-Akt-mTOR-HIF1alpha pathway. Dev Comp Immunol.

[CR18] Hurt AC (2014). The epidemiology and spread of drug resistant human influenza viruses. Curr Opin Virol.

[CR19] Iuliano AD, Roguski KM, Chang HH, Muscatello DJ, Palekar R, Tempia S, Cohen C, Gran JM, Schanzer D, Cowling BJ, Wu P, Kyncl J, Ang LW, Park M, Redlberger-Fritz M, Yu H, Espenhain L, Krishnan A, Emukule G, van Asten L, Pereira da Silva S, Aungkulanon S, Buchholz U, Widdowson MA, Bresee JS, Global Seasonal Influenza-associated Mortality Collaborator N (2018) Estimates of global seasonal influenza-associated respiratory mortality: a modelling study. Lancet 391:1285–1300 10.1016/S0140-6736(17)33293-2 PMC5935243 29248255

[CR20] Iwasaki A, Pillai PS (2014). Innate immunity to influenza virus infection. Nat Rev Immunol.

[CR21] Koyuncu E, Purdy JG, Rabinowitz JD, Shenk T (2013). Saturated very long chain fatty acids are required for the production of infectious human cytomegalovirus progeny. PLoS Pathog.

[CR22] Levy PL, Duponchel S, Eischeid H, Molle J, Michelet M, Diserens G, Vermathen M, Vermathen P, Dufour JF, Dienes HP, Steffen HM, Odenthal M, Zoulim F, Bartosch B (2017). Hepatitis C virus infection triggers a tumor-like glutamine metabolism. Hepatology.

[CR23] Li TC, Chan MC, Lee N (2015). Clinical Implications of Antiviral Resistance in Influenza. Viruses.

[CR24] Matrosovich M, Matrosovich T, Garten W, Klenk HD (2006). New low-viscosity overlay medium for viral plaque assays. Virol J.

[CR25] Mazzon M, Castro C, Thaa B, Liu L, Mutso M, Liu X, Mahalingam S, Griffin JL, Marsh M, McInerney GM (2018). Alphavirus-induced hyperactivation of PI3K/AKT directs pro-viral metabolic changes. PLoS Pathog.

[CR26] Michelakis ED, Webster L, Mackey JR (2008). Dichloroacetate (DCA) as a potential metabolic-targeting therapy for cancer. Br J Cancer.

[CR27] Miyake Y, Ishii K, Honda A (2017). Influenza virus infection induces host pyruvate kinase M which interacts with Viral RNA-dependent RNA polymerase. Front Microbiol.

[CR28] Muller KH, Kakkola L, Nagaraj AS, Cheltsov AV, Anastasina M, Kainov DE (2012). Emerging cellular targets for influenza antiviral agents. Trends Pharmacol Sci.

[CR29] Passalacqua KD, Lu J, Goodfellow I, Kolawole AO, Arche JR, Maddox RJ, Carnahan KE, O'Riordan MXD, Wobus CE (2019). Glycolysis is an intrinsic factor for optimal replication of a norovirus. MBio.

[CR30] Pelicano H, Martin DS, Xu RH, Huang P (2006). Glycolysis inhibition for anticancer treatment. Oncogene.

[CR31] Petiot E, Jacob D, Lanthier S, Lohr V, Ansorge S, Kamen AA (2011). Metabolic and kinetic analyses of influenza production in perfusion HEK293 cell culture. BMC Biotechnol.

[CR32] Ren L, Zhang W, Han P, Zhang J, Zhu Y, Meng X, Zhang J, Hu Y, Yi Z, Wang R (2019). Influenza A virus (H1N1) triggers a hypoxic response by stabilizing hypoxia-inducible factor-1alpha via inhibition of proteasome. Virology.

[CR33] Ritter JB, Wahl AS, Freund S, Genzel Y, Reichl U (2010). Metabolic effects of influenza virus infection in cultured animal cells: intra-and extracellular metabolite profiling. BMC Syst Biol.

[CR34] Sanchez EL, Lagunoff M (2015). Viral activation of cellular metabolism. Virology.

[CR35] Schmittgen TD, Livak KJ (2008). Analyzing real-time PCR data by the comparative C(T) method. Nat Protoc.

[CR36] Semenza GL (2012). Hypoxia-inducible factors in physiology and medicine. Cell.

[CR37] Smallwood HS, Duan S, Morfouace M, Rezinciuc S, Shulkin BL, Shelat A, Zink EE, Milasta S, Bajracharya R, Oluwaseum AJ, Roussel MF, Green DR, Pasa-Tolic L, Thomas PG (2017). Targeting metabolic reprogramming by influenza infection for therapeutic intervention. Cell Rep.

[CR38] Thai M, Graham NA, Braas D, Nehil M, Komisopoulou E, Kurdistani SK, McCormick F, Graeber TG, Christofk HR (2014). Adenovirus E4ORF1-induced MYC activation promotes host cell anabolic glucose metabolism and virus replication. Cell Metab.

[CR39] Thai M, Thaker SK, Feng J, Du Y, Hu H, Ting WuT, Graeber TG, Braas D, Christofk HR (2015). MYC-induced reprogramming of glutamine catabolism supports optimal virus replication. Nat Commun.

[CR40] Varanasi SK, Rouse BT (2018). How host metabolism impacts on virus pathogenesis. Curr Opin Virol.

[CR41] Wang GL, Semenza GL (1993). General involvement of hypoxia-inducible factor 1 in transcriptional response to hypoxia. Proc Natl Acad Sci U S A.

[CR42] Wang GL, Semenza GL (1995). Purification and characterization of hypoxia-inducible factor 1. J Biol Chem.

[CR43] Wolf A, Agnihotri S, Micallef J, Mukherjee J, Sabha N, Cairns R, Hawkins C, Guha A (2011). Hexokinase 2 is a key mediator of aerobic glycolysis and promotes tumor growth in human glioblastoma multiforme. J Exp Med.

[CR44] Xu C, Liu X, Zha H, Fan S, Zhang D, Li S, Xiao W (2018). A pathogen-derived effector modulates host glucose metabolism by arginine GlcNAcylation of HIF-1alpha protein. PLoS Pathog.

[CR45] Yogev O, Lagos D, Enver T, Boshoff C (2014). Kaposi's sarcoma herpesvirus microRNAs induce metabolic transformation of infected cells. PLoS Pathog.

[CR46] Yu Y, Maguire TG, Alwine JC (2014). ChREBP, a glucose-responsive transcriptional factor, enhances glucose metabolism to support biosynthesis in human cytomegalovirus-infected cells. Proc Natl Acad Sci U S A.

[CR47] Zhang W, Wang G, Xu ZG, Tu H, Hu F, Dai J, Chang Y, Chen Y, Lu Y, Zeng H, Cai Z, Han F, Xu C, Jin G, Sun L, Pan BS, Lai SW, Hsu CC, Xu J, Chen ZZ, Li HY, Seth P, Hu J, Zhang X, Li H, Lin HK (2019) Lactate Is a natural suppressor of RLR signaling by targeting MAVS. Cell 178:176–189 10.1016/j.cell.2019.05.003 PMC6625351 31155231

[CR48] Zhao Y, Chahar HS, Komaravelli N, Dossumekova A, Casola A (2019). Human metapneumovirus infection of airway epithelial cells is associated with changes in core metabolic pathways. Virology.

[CR49] Zhao Y, Wang A, Zou Y, Su N, Loscalzo J, Yang Y (2016). *In vivo* monitoring of cellular energy metabolism using SoNar, a highly responsive sensor for NAD(+)/NADH redox state. Nat Protoc.

[CR50] Zumla A, Rao M, Wallis RS, Kaufmann SHE, Rustomjee R, Mwaba P, Vilaplana C, Yeboah-Manu D, Chakaya J, Ippolito G, Azhar E, Hoelscher M, Maeurer M (2016). Host-directed therapies for infectious diseases: current status, recent progress, and future prospects. Lancet Infect Dis.

