# Supplementary material for: Influenza A Virus (H1N1) Infection Induces Glycolysis to Facilitate Viral Replication
Source: Virol Sin. 2021 Sep 14;36(6):1532–42. doi: 10.1007/s12250-021-00433-4 (PMC8692537; doi:10.1007/s12250-021-00433-4)
Supplement: Supplementary file 1 — Supplementary file1 (PDF 146 KB) [file 12250_2021_433_MOESM1_ESM.pdf]

VIROLOGICA SINICA

Electronic Supplementary Material

Influenza A Virus (H1N1) Infection Induces Glycolysis to Facilitate Viral Replication

Lehao Ren[1,2#] • Wanju Zhang[3#] • Jing Zhang[4#] • Jiaxiang Zhang[1] • Huiying Zhang[5] • Yong Zhu[1] • Xiaoxiao Meng[1] • Zhigang Yi[5✉] • Ruilan Wan[1✉]

1. Department of Emergency and Critical Care Medicine, Shanghai General Hospital, Shanghai Jiao Tong University School of Medicine, Shanghai 201620, China
2. Department of Critical Care Medicine, Union Hospital, Tongji Medical College, Huazhong University of Science and Technology, Wuhan 430022, China
3. Microbiology Laboratory, Shanghai Municipal Centre for Disease Control and Prevention, Shanghai 200336, China
4. Department of Pathology, Zhongshan Hospital, Fudan University, Shanghai 200032, China
5. Department of Pathogen Diagnosis and Biosafety, Shanghai Public Health Clinical Center, Fudan University, Shanghai 201508, China

Supporting information to DOI: 10.1007/s12250-021-00433-4

Table S1 The primer sequences used in qRT-PCR

Target genes	Sequence
HIF-1α	F: 5'-CGCAAGTCCTCAAAGCACAG-3'<br>R: 5'-TCATCAGTGGTGGCAGTGGT-3'
IFN-α	F: 5'-ATCTCTCCTTCCTCCTGTCT-3'<br>R: 5'-TTGCCATCAAACTCCTCCT-3'
IFITM1	F: 5'-TCTTCTTGAACTGGTGCTGTC-3'<br>R: 5'-GTCGCGAACCATCTTCCTGT-3'
ISG56	F: 5'-TAGCCAACATGTCCTCACAGAC-3'<br>R: 5'-TCTTCTACCACTGGTTTCATGC-3'
MxA	F: 5'-CCACTGGACTGACGACTTGA-3'<br>R: 5'-GAGGGCTGAAAATCCCTTTC-3'
viral RNA (*M* gene)	F: 5'-GACCRATCCTGTCACCTCTGAC-3'<br>R: 5'-AGGGCATTYTGGACAAAKCGTCTA-3'
β-actin	F: 5'-AAGGTGACAGCAGTCGGTT-3'<br>R: 5'-TGTGTGGACTTGGGAGAGG-3'

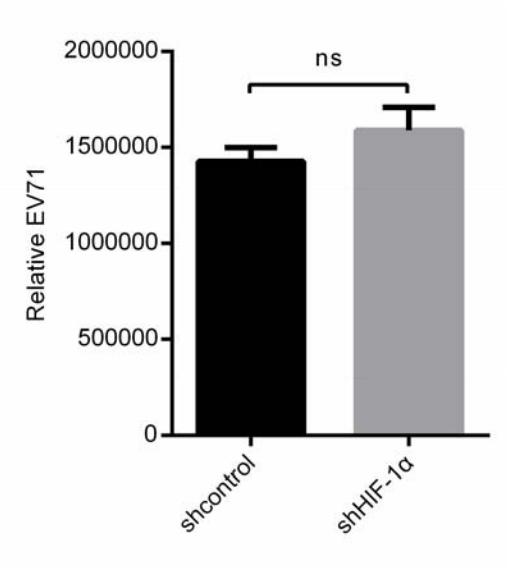

Fig. S1 Viral replication of EV71 is not impaired by knocking down HIF-1α in A549 cells. Shcontrol A549 and shHIF-1α A549 cells were infected with luciferase labeled EV71 (N-Luc EV71) virus. At 24 h p.i., cells were lysed to measure the relative light units (RLUs) in a luminometer. ns, not significant.
